# An Historical View of Surgery in the Sixteenth Century

**Published:** 1799-11

**Authors:** 


### *An Hiſtorical View of Surgery in the Sixteenth Century.*

[ Continued from our laſt Number, pp. 278—281.]

§ 15. AMONG the moſt learned ſurgeons of that century, we may doubtleſs mention FELIX WUERZ, of Baſel, whoſe Manual of Surgery, in German, is ſo judiciouſly written for the age in which he lived, and contains ſo many excellent principles, never before developed, that even modern ſurgeons might derive much information from this elementary work. His valuable Treatiſe on concealed Ruptures deſerves particular notice among the multiplicity of ſubjects, as it is the only diſſertation of the kind we poſſeſs. He alſo controverts, in a variety of paſſages, the prevailing prejudices of his cotemporaries, condemns the ſtitching up of wounds, as well as cauterization in hemorrhages, while he diſapproves of the frequent examination of ulcers by the probe, and the uſe of dilators.

FRANC. DE ARCE, of Frejenal in Seville, who practiſed ſurgery at Llerena and Valverde, in Eſtremadura, was ſo celebrated for the cure of fiſtulous ulcers, that patients from all parts of France, Italy, and Spain came to him in numbers, for the cure of thoſe troubleſome complaints. He principally uſed gum guajacum, and, inſtead of the uſual oleaginous remedies, he recommended a balſam of his own invention, known by the name of *Balſamus Arcaei*. In malignant ſores, he applied the actual cautery, and recommended the uſe of the trepan; which, however, in very ſevere and extenſive fractures of the bones, and particularly in very young children, he conſidered as an unneceſſary operation, becauſe in theſe the fractured bones frequently re-unite without the aid of art.*

The

---

* FRANC. ARCEUS, *a moſt excellent and compendious method of curing woundes in the head and in other partes of the body:* Tranſlated by J. READ. Vol. II. c. 2. f. 29. b. Quarto. London 1588. This Tranſlation is very little known, and is not even mentioned by HALLER. Prof. SPRENGEL obtained a copy of it from Prof. MECKEL, the Anatomiſt of Halle.

The celebrated anatomist, JUL. CÆSAR. ARANZI, Professor at Bologna, wrote a Treatise on Ulcers, in which he first attempted to describe the distortion of the penis arising from a varicose dilatation of the vessels, after too frequent coition.* In hydrocephalus he bestows much praise on the external application of the *Empl. diapalma*, which produces a warm and general perspiration. The saccharine acid obtained from honey is, according to him, a mild caustic for removing the pterygium. He farther invented a peculiar forceps for the extirpation of nasal polypi, and was uncommonly successful in the operation for fistul vasculares. On the contrary, he treated aneurisms merely with astringent remedies, without the operation; and for the cure of cancerous ulcers he simply suggested mild remedies, such as the marsh-mallow root and oil of almonds.

§ 16. AMBROSIUS PARE' was unquestionably the most celebrated surgeon of the 16th century. He was a native of Laval, in the Province of Maine, had served as surgeon in several wars, particularly in the third campaign of Francis I. against the Emperor in Italy, and he likewise accompanied Henry II. in the battles near Renti and S. Quentin. He was afterwards successively first surgeon to the French Kings Francis II. and Charles IX. He gained the confidence of the latter to such a degree that he was the only person spared, and for whose safety the King was personally interested during the massacre of the Huguenots at Paris, in the well-known night of St. Bartholomew. PARE' evinced his gratitude to the king by the most solicitous attention to his health, and by so strict and honourable a fidelity, that even after the decease of that monarch he expressed himself with a prudent and becoming reserve, concerning the cause of his premature death.

Besides the improved treatment of gunshot-wounds, which he had the merit of introducing, together with many other peculiar methods in operative surgery, he has rendered essential service to different branches of that science. He treated, for instance, the hydrocele with a seton; as the dangerous consequences of incision were in that age more frequently observed than they are at present. He did not apply the actual cautery to wounded blood-vessels, according to the old practice, but secured them by the ligature. The fracture of the collum ossis femoris, formerly considered as a luxation of that bone, was first ascertained by him with accuracy: he also reprobated the frequent dressing of ulcers, and the application of the trepan to the sutures of the cranium and the temporal bones

* AURANTIUS *de tumoribus præternaturalibus*. C. 50. p. 245.—4to. Venet. 1595.

bones. He made very judicious remarks on concuſſions of the brain, of which Henry II. died, and on ſuppurations of the liver, ariſing from injuries of the head. Wounds of the throat, in which one of the jugular veins, and even the trachea was cut through, he did not conſider as mortal. He ſuccefsfully treated an injury of the nervus medianus from veneſection, and thereby acquired the confidence of Charles IX. who had been ſubject to that dangerous accident. A perſon who, from loſing a great part of his tongue, had been ſpeechleſs for a conſiderable time, accidentally recovered the power of ſpeech, by thruſting a table-ſpoon into his mouth: PARE′ ingeniouſly imitated this method, by contriving an appropriate inſtrument.

§ 17. JACOB GUILLEMEAU, of Orleans, firſt ſurgeon to Henry the Great, and ſuperintendant of the Hôtel-Dieu, was a pupil of PARE′, and obtained reputation in the chirurgical world, chiefly by his improvements of the trepan. In order to prevent any injury of the brain and its membranes, in the application of the inſtrument, he fixed a *chapperon* under the crown of the trepan, by which its deſcent on the membranes of the brain ſhould be checked; or he cauſed the crown of that inſtrument to be circularly indented, that the teeth may be uniformly applied to the bone, and thus prevent its deſcending farther than is neceſſary. This improvement was cenſured by JOH. PETER PASSERO, a ſurgeon of Bergamo; becauſe the circumference of the bone was thereby made too rough to favour a ſpeedy re-production of new ſubſtance: this addition to the trepan has, however, in later times, been almoſt generally adopted. But the *chapperon* of GUILLEMEAU is, in reality, an inconvenient and uſeleſs part of the inſtrument inſomuch that even after the late improvements attempted by KLINDWORD, it cannot be employed with advantage. The former made uſe of the exfoliating trepan in thoſe caſes where only the upper table of the cranium was injured, and where it became neceſſary to remove the blood ſtagnating in the *diploe*. When the *dura mater* was laid bare, and the pus could be freely diſcharged, GUILLEMEAU conſidered the application of the trepan altogether unneceſſary. The veſſels diſſected in amputation, if accompanied with ſphacelated parts, he treated with the actual cautery; while, in the contrary caſe, he applied the ligature. The paracenteſis he performed at the diſtance of three fingers breadth below the umbilicus, in a lateral direction; which, however, in ſome caſes, might be too near the navel. He deviated from the principles of his teacher in performing a radical cure of the hydrocele, and preferred the inciſion into the *tunica vaginalis teſtis* to the ſeton and the cauſtic. Another cotemporary ſurgeon,

FRANCO, on the contrary, reforted to the *point doré*, or the lacing up the fwollen parts with golden threads, without injuring the feminal veffels. GUILLEMEAU was an excellent operator for aneurifms, and he removed varicofe nodules with cauftics, for which he particularly recommended his favourite *cautere de velours*, prepared from the lees of foap-boilers; while FRANCO employed the actual cautery even in varicocele. In necrofis, Guillemeau likewife applied this violent remedy.

JOH. TAGAULT, of Amiens, who taught furgery both at Paris and Padua, is the author of a Compendium,* which, however, confifts chiefly of an improved edition of that previoufly publifhed by GUY OF CHAULIAC.

§ 18. JOH. PHILIP INGRASSIAS, of Rachalbuto, in Sicily, was a great anatomift who fucceffively filled the Profefforial Chair at Padua, Naples, and Palermo, and was appointed, by King Philip II. director of medicinal affairs in the Two Sicilies. He wrote a fyftematic work on ulcers, in which he added to the fixty-one kinds defcribed by GALEN, 165 new fpecies; but among thefe he included many chirurgical difeafes which were improperly placed in that clafs. Among other errors, we may notice an obfervation occurring in his "*Iatropologia*," p. 170 (8vo. Panorm. 1546), in which he confiders a fracture of the trochanter as a fimple luxation. In the capacity of fuperintendant of medical affairs, he fo far reftricted the free exercife of the chirurgical art, that furgeons were obliged to proceed in their method of cure confiftently with the indications formed by phyficians. The caufes of this reftriction he fully explained in a particular work, where, among other modes of treatment peculiar to himfelf, he advifes to perform amputation in the mortified part, and to cauterize the found contiguous parts. He alfo wrote the hiftory of the difeafe, of which he cured the Duke of Terranuova, when feveral phyficians of great eminence were confulted, whofe opinions he communicated in a work, entitled, "*Ducis Terranovæ cafus enarratio et curatio;*" 4to. Venet. 1568. The difeafe here alluded to was a fracture of the ribs combined with an empyema, for which INGRASSIAS fuccefsfully employed the guajacum and cauftics.

JOH. BAPT. CARCANO LEONE, of Milan, and Profeffor in Pavia, wrote a very imperfect work on wounds of the head, though he was a pupil not altogether unworthy of the great FALLOPIUS. Befides other proofs of his defective judgment, this work contains inftructions for dividing

* *Tagaultii de Chirurgica Inftitutione, lib.* vi. 8vo. Venet. 1549.

viding fractures of the cranium by wooden wedges, to promote the evacuation of pus. He raised the depressed bones with instruments; and cautions the reader against the application of the trepan. The removal of a part of the cortical substance of the brain is to him a dreadful attempt, and he at length ingenuously acknowledges, that he never succeeded in curing wounds of the head.

The theory and practice of curing the diseases of the eye were very little improved in that century; though towards its close there appeared a peculiar and celebrated work on the disorders of that organ, by George Bartisch, of Königsbrück, oculist to the Electoral Court of Saxony. His propensity to superstitious opinions was so great, that he recommended strict attention to the influence of the planets, before any operations on the eyes could be undertaken with safety, or advantage. His theory of the cataract he had borrowed from the Arabian writers; and he admitted the existence of a film or web, which descends from the brain into the aqueous humour of the eye. According to him, there are five species of cataract; namely, the white, grey, blue, green, and yellow. He also asserts, that he has frequently observed the gutta serena to be an hereditary disease; and in every instance he advises the practitioner to puncture or press it down with a perfectly straight conical needle. For the cure of *ptosis*, he proposes an instrument, by which the skin of the eye-lid is compressed between two plates. Verduyn has made an improvement on this instrument.

[ To be concluded in our next Number. ]

